# Factors of suicidal behavior among inpatients with major depressive disorder: A retrospective case series

**DOI:** 10.3389/fpsyt.2022.996402

**Published:** 2022-09-23

**Authors:** Chaomeng Liu, Weigang Pan, Dandi Zhu, Fanqiang Meng, Tengfei Tian, Li Li, Xiaohong Li

**Affiliations:** ^1^The National Clinical Research Center for Mental Disorders and Beijing Key Laboratory of Mental Disorders, Beijing Anding Hospital, Capital Medical University, Beijing, China; ^2^Advanced Innovation Center for Human Brain Protection, Capital Medical University, Beijing, China

**Keywords:** suicidal behavior, binary logistic regression, major depressive disorder, matching of the propensity score, case series

## Abstract

**Objective:**

Previous studies indicate that more than half of those who died by suicide had a depressive disorder. When discussing the factors associated to suicidal behavior (SB) among patients with major depressive disorder (MDD), sociocultural contexts should also be carefully considered. This case series study explored the factors correlated to SB among MDD patients in Beijing, China.

**Methods:**

The patient information sheets were retrieved from an electronic database that comprised patient medical information. Three forms of binary logistic regression equations were conducted to explore the factors associated to SB among patients with MDD. For the inconsistent variables produced by the three regression models, the propensity score matching (PSM) analysis was done for further verification.

**Results:**

In this retrospective study, 1,091 depressed cases were enrolled. The difference between the SB group and non-SB group in gender, impulsivity, the severity of depression, history of major mental trauma, and family history of suicide were statistically significant in univariate comparisons (*P* < 0.05); the binary logistic regression analysis and the PSM analysis showed that female gender, history of major mental trauma, impulsivity, family history of suicide and severity of depression were factors correlated to SB among patients with MDD (odds ratios >1).

**Conclusions:**

Female gender, the history of major mental trauma, impulsivity, the severity of depression, and family history of suicide were independently associated with the appearance of SB among MDD patients in Beijing, China. Inevitably, these findings should be viewed with particular caution due to the inherent drawbacks of a retrospective nature. More prospective longitudinal research should be conducted to examine those dynamic alterations in the corresponding confounders.

## Introduction

Suicide causes almost 800,000 deaths worldwide, representing 1.5% of the total death toll, with severe consequences for global public health (1). Studies indicate that about 60% of those who died by suicide had a major depressive disorder (MDD), which negatively affects how people think, feel, and act (2, 3). About 50% of people who die of suicide visit their general practitioners within 3 months prior to their deaths, 40% within 1 month, and 20% within 1 week before death (4, 5), indicating the important role of primary care in preventing suicide in this group.

Depression is one of the most frequently seen diseases in public. Most people with MDD have a certain amount of stigma and are reluctant to share their situation with others, which greatly adds to the difficulty in detecting high-risk suicidal individuals (6). In this scenario, it is important to identify the factors associated to suicidal behavior (SB), including suicidal ideation and suicide attempt, among people with MDD, for clinicians to recognize high-risk people and make appropriate interventions, as the suicide rate is reported to be reduced by close monitoring and aggressive treatment (7). Unfortunately, the link between SB-related factors among MDD patients and suicidality is frequently under-reported. For example, more than 2% of traffic accidents are closely related to SB. However, the phenomenon may be underreported, considering that suicides from car accidents may be reported as accidental in national statistics (8). Depression has previously been suggested to be closely related to suicide, but depression severity alone cannot accurately predict SB (9). Consequently, identifying more SB-related factors among depression patients is of great significance.

A previous meta-analysis explored SB-related risk factors among patients with MDD, and the results showed that male gender, prior suicidal attempts, family history of psychiatric disorder, the severity of depression, concurrent diseases, and hopelessness were closely related to SB (4). SB is an outcome of complex impacts of psychotic, mental, physical, cultural, and social factors; thus, determining the correlation factors of SB is difficult. In the present case, 44% of those who commit suicide are 65 or older; this rate is much higher than 18% in the United States (10, 11). Some adverse social and cultural impacts on older adults in rural China were suggested to be a key factor contributing to SB (12). Therefore, when discussing the factors correlated to SB among MDD patients, the sociocultural contexts should also be carefully considered (13).

In terms of society, ~1/4 (24%) of inpatients who commit suicide occur 3 months after discharge from psychiatric hospitals (14). The suicide risk prediction model may be an efficient way to assess the risk of suicide. It uses inpatient medical records and is more accurate than the prediction based on clinical judgment (15). The present preliminary case series study retrospectively explored the factors associated to SB with the help of inpatient medical records through propensity score matching (PSM) and logistic regression analysis. Here, we hypothesized that the factors correlated to SB among MDD patients in Beijing, China, differ from those in other countries due to the difference in sociocultural contexts.

## Materials and methods

### Study design and data sources

The present case series study retrospectively collected consecutive cases at Beijing Anding Hospital (Beijing, China) between November 1st 2017 and December 31st 2018. The medical records of the patients were obtained using the International Classification of Diseases, Tenth Revision (ICD-10) code in a clinical electronic database comprising the medical data of the patients, such as demographics, outpatient diagnoses, inpatient management, medical procedure, drug prescription and discharge diagnosis (16). The first two authors independently extracted the data information closely related to this study, and inconsistencies were resolved through consultation with the third author. The patients hospitalized repeatedly during the study were excluded to avoid duplicated or over-presented information from individual patients.

### Study object and case definition

Depression cases with age 18 or older and < 60 years recorded in the medical records were recruited. Among them, depression was diagnosed following the ICD-10 disease codes in primary/secondary diagnoses, including F32 (depressive episode), F32.2 (the serious depressive episode without any psychotic symptom), F32.3 (the serious depressive episode in the presence of psychotic symptoms), F33 (relapsed depression disease), F33.2 (relapsed depression disease and/or present serious episode without any psychotic symptom), and F33.3 (relapsed depression disease, and present serious episode in the presence of psychotic symptoms). The index data were defined as the date when depression was diagnosed. Furthermore, depressive symptoms may occur in the preclinical stage of dementia (17). Therefore, this study ruled out dementia cases diagnosed (ICD-10 code F00-F03) 2 years after the index date. Finally, the present retrospective work enrolled 1,091 cases of depression.

### Covariates

The collected data included gender, age, marital status, hobbies, monthly income, quality of interpersonal relationships, impulsivity, first episode or recurrence, the severity of depression and with/without somatic diseases, history of major mental trauma, drinking, and smoking, as well as family histories of suicide, psychiatric disorder, and SB, including suicidal ideation and suicide attempts. Among them, age groups were classified as groups 18–29, 30–39, 40–49, or 50–59-year old groups; the marital status of patients in married, unmarried, widowed, or divorced; the monthly income into <1,000, 1,000–3,000, 3,001–5,000, and >5,000 RMB (¥); interpersonal relation quality into good, general or poor. Furthermore, the Hamilton Rating Scale (HAMD, with 17 items) was adopted to assess the severity of depression, while suicidal attempts and suicidal ideation were evaluated mainly through in-person interviews by clinicians, according to the 3rd item in HAMD-17. In this study, SB was adopted as the dependent variable, and the others were used as independent variables based on previous findings and practical experience (4, 18, 19).

### Data processing and statistical analysis

Statistical analysis was completed with SPSS 26.0 (Chicago, IL, USA) with a significance level of 5%. Prior to the analysis of the inferential data, we determined the normality of the data and the missing data. The categorical descriptive variables (like age, gender) were presented as counts and percentages. In addition, we used the Chi-square test to compare unordered categorical variables and the Wilcoxon test to compare ordered categorical variables between the group of patients with or without SB. Then we carried out a univariate analysis to identify independent variables for SB.

Binary logistic regression analysis helps to determine the independent variables associated with SB in depression cases. To find predicting variables for the regression equation more accurately, this study adopted three common regression models, including univariate, enter, and stepwise filtering (Forward LR) (18, 20, 21). In addition, regression equation validity was validated by the Hosmer-Lemeshow (HL) goodness-of-fit test; the idea of the HL test is to partition the observations into groups and construct a chi-squared statistic that summarizes the discrepancy between the number of observed and expected events within all combinations of group and outcome state. When the accompanying *p*-value is >0.05, it usually means a good fit effect of the regression equation (22). For unordered multiclass variables, such as marital status, set them as dummy variables in the regression models. The odds ratios (OR) and the relevant 95% confidence intervals (CI) were obtained to test the fitness of the condition within the regression equation, and OR values >1 indicated risk factors; correspondingly, OR values <1 represented protective factors.

Uncertain SB-related independent variables obtained from three logistic regression models were verified by PSM. PSM analysis is an ideal matching approach, which was considered to maximally reduce selection bias within retrospective studies while achieving a similar effect to that of a randomized controlled study (23). The matched analysis 1:1 according to the closest-neighbor matching with the 0–1 tolerance should be done according to the estimated PSM scores (24). For the two groups, those matched variables were further verified by the equilibrium test.

## Results

### Univariate comparison

The results of the univariate comparison are listed in Table 1. Differences in genders, the severity of depression, impulsivity, history of major mental trauma, and family history of suicide were statistically significant between the non-SB and SB groups. Specifically, the proportion of women in the patient group with SB increased markedly compared to those without SB, as well as in the aspects of impulsivity, the severity of depression, family history of suicide, and history of major mental trauma. There were no significant differences in age, monthly income, marital status, hobbies, interpersonal relation quality, first episode or recurrence, with/without somatic diseases, histories of drinking and smoking, as well as family history of psychiatric disorder between non-SB and SB groups.

**Table 1:** Characteristics of depression cases with or without suicidal behavior.

**Characteristics**	**Patients with SB**	**Patients without SB**	***P*-value**
	***n* = 405**	***n* = 686**	
Age at index date (years)			0.637
18–29	131 (32.3%)	185 (27.0%)	
30–39	68 (16.8%)	154 (22.4%)	
40–49	85 (21.0%)	154 (22.4%)	
50–59	121 (29.9%)	193 (28.1%)	
Gender (female)	298 (73.6%)	458 (66.8%)	0.018
Marital status			0.468
Unmarried	125 (30.9%)	189 (27.6%)	
Married	249 (61.5%)	432 (63.0%)	
Divorced	26 (6.4%)	58 (8.5%)	
Widowed	5 (1.2%)	7 (1.0%)	
Monthly income (RMB, ¥)			0.500
<1,000	25 (6.2%)	38 (5.5%)	
1,001–3,000	123 (30.4%)	199 (29.0%)	
3,001–5,000	121 (29.9%)	209 (30.5%)	
>5,000	136 (33.6%)	240 (35.0%)	
Quality of interpersonal relations			0.309
Poor	20 (4.9%)	42 (6.1%)	
General	129 (31.9%)	230 (33.5%)	
Good	256 (63.2%)	414 (60.3%)	
Hobbies	36 (8.9%)	64 (9.3%)	0.808
Impulsivity	114 (28.1%)	99 (14.4%)	0.000
First episode	158 (39.0%)	283 (41.3%)	0.466
Severity of depression			0.000
Mild	6 (1.5%)	38 (5.5%)	
Moderate	36 (8.9%)	126 (18.4%)	
Severe	363 (89.6%)	522 (76.1%)	
Somatic diseases	300 (74.1%)	491 (71.6%)	0.372
Smoking	78 (19.3%)	123 (17.9%)	0.584
Drinking	50 (12.3%)	80 (11.7%)	0.736
Major mental trauma history	62 (15.3%)	74 (10.8%)	0.029
Family history of suicide	35 (8.6%)	35 (5.1%)	0.021
Family history of psychiatric disorder	123 (30.4%)	224 (32.7%)	0.434

Hobbies: number of hobbies; First episode: number of patients with first-episode depression; Somatic diseases: number of patients with somatic diseases; Smoking: number of people who have a history of smoking; Drinking: number of people who have a history of drinking.

### Binary logistic regression analysis

Independent variables associated with SB among depression cases were explored by binary logistic regression after adjusting for confounders (Table 2). As revealed by the HL test, all three regression models showed a favorable fitting effect (All *P*-values were >0.05). In model 1, the female gender, family history of suicide, major mental trauma history, the severity of depression, and impulsivity were taken into the regression equation, which implied that the female gender, family history of suicide, history of major mental trauma, the severity of depression, and impulsivity were factors correlated to the occurrence of SB. Similarly, in Model 2, we included major mental trauma history, depression severity, and impulsivity into the regression equation. In particular, in model 3, the critical independent variables incorporated into the regression equation were the same as those in model 2.

**Table 2:** Factors associated to the appearance of suicidal behavior among patients with MDD.

**Methods**	**HL test *P*-value**	**Independent variables**	**OR, 95% CI**	***P*-value**
Model 1	0.787	Female gender	1.492 (1.062–2.097)	0.021
		Family history of suicide	1.820 (1.044–3.174)	0.035
		Major Mental Trauma History	1.548 (1.052–2.277)	0.027
		Impulsivity	2.224 (1.617–3.059)	0.000
		Severity of depression	2.294 (1.671–3.150)	0.000
Model 2	0.837	Major Mental Trauma History	1.571 (1.079–2.286)	0.018
		Impulsivity	2.294 (1.684–3.125)	0.000
		Severity of depression	2.288 (1.676–3.123)	0.000
Model 3	0.673	Major Mental Trauma History	1.517 (1.039–2.215)	0.031
		Impulsivity	2.233 (1.635–3.049)	0.000
		Severity of depression	2.219 (1.624–3.033)	0.000

Model 1: enter; Model 2: stepwise; Model 3: univariate filtering; HL test: Hosmer-Lemeshow Test; statistical test differences were considered significant if the P-values were < 0.05.

### PSM analysis

It can be clearly seen from Table 2 that the same independent variables selected from the three regression models were a history of major mental trauma, impulsivity, and severity of depression. Compared to Models 2 and 3, there are two more independent variables, namely female gender and family history of suicide. Suicide is highly familial and has been widely documented around the world. Therefore, it remains unclear whether the female gender was the factor that independently predicted the risk of SB among cases of depression. To better validate the associations of the female gender with SB, the 1,091 cases were classified as two groups according to gender (335 men and 756 women), with age, monthly income, marital status, hobbies, quality of interpersonal relationships, first episode or recurrence, with/without somatic diseases, drinking and smoking being predictive variables in the PSM analysis. A match tolerance of 0.005 was established during the SPSS analysis, without replacement of sampling; meanwhile, the above predictive variables were appropriately balanced according to the PSM analysis between the men's and women's groups. As seen in Table 3, a total of 199 of 335 male patients were matched among 756 female patients, and the female gender remained a factor in independently predicting the risk of SB in cases of depression (*P* = 0.000).

**Table 3:** Characteristics of cases of depression of different genders by propensity score matching.

**Characteristics**	**Male patients with depression**	**Female patients with depression**	***P*-value**
	***n* = 199**	***n* = 199**	
Age at index date (years)			0.391
18–29	83 (41.7%)	93 (46.7%)	
30–39	43 (21.6%)	32 (16.0%)	
40–49	34 (17.1%)	29 (14.6%)	
50–59	39 (19.6%)	45 (22.6%)	
Marital status			0.780
Unmarried	89 (44.7%)	91 (45.7%)	
Married	98 (49.2%)	96 (48.2%)	
Divorced	11 (5.5%)	12 (6.0%)	
Widowed	1 (0.5%)	0 (0.0%)	
Monthly income (RMB, ¥)			0.076
<1,000	13 (6.5%)	5 (2.5%)	
1,001–3,000	55 (27.6%)	58 (29.1%)	
3,001–5,000	58 (29.1%)	75 (37.7%)	
>5,000	73 (36.7%)	61 (30.7%)	
Quality of interpersonal relations			0.298
Poor	13 (6.5%)	19 (9.5%)	
General	86 (43.2%)	73 (37.7%)	
Good	100 (50.3%)	107 (53.8%)	
Hobbies	16 (8.0%)	16 (8.0%)	1.000
First episode	84 (42.2%)	86 (43.2%)	0.417
Somatic diseases	142 (71.4%)	127 (63.8%)	0.108
Smoking	41 (20.6%)	43 (21.6%)	0.806
Drinking	26 (13.1%)	28 (14.1%)	0.770
Suicidal behavior	61 (30.7%)	95 (47.7%)	0.000

## Discussion

The present case series study retrospectively indicated that female gender, history of major mental trauma, impulsivity, the severity of depression, and family history of suicide were factors associated to SB among adult depression cases based on electronic medical records in Beijing, China. Inevitably, these findings should be viewed with particular caution due to the inherent drawbacks of a retrospective nature.

Many articles investigate the association of gender with suicide-related behaviors, including suicide (which refers to suicide death), suicide attempts, and suicidal ideation. Although in most countries, suicide rates in males are higher than those in females, the prevalence of SB (suicide attempts and suicidal ideation) in females is higher than that in males in almost all countries (4, 14, 25). Thus, depressed inpatients with SB were more likely being females in our study. It is worth noting that the male-female ratios of suicide rates in China were ~1:1, although the gender ratio increased in the last two decades, and these gender ratios were almost 3:1 in most other countries (26, 27). For this, there may be two main reasons. First, there are about five million people in the rural areas of Beijing, and numerous females in the rural areas show a poor sociocultural status. In terms of suicide means, since pesticides are available in almost all families, taking lethal pesticides is often the first choice to commit suicide (28). As a result of being less economically developed compared to urban areas, pesticide poisoning is often not effectively treated, leading to higher suicide rates. Second, rural women are generally less educated and lack effective means of coping with negative events in their lives. Coupled with the fact that there are relatively few mental health services in rural areas, the access for rural women with psychological distress to psychotherapy is relatively poor (29).

Expectedly, a major history of mental trauma increases the risk of suicide; patients with unfavorable life experiences were associated with a higher risk of SB (30). The types of primary mental trauma are different in various stages of life. The experience of bullying is one of the main trauma associated with SB in children. When you grow up, there are more traumas that trigger SB intention, with emotional trauma being the main cause of adolescent SB. Typically, emotional and physical loss of family is the main factor related to the risk of SB in young adults (31). In fact, hypothalamic-pituitary-adrenal (HPA) axis dysfunction has previously been observed, which stimulates the adrenal cortex to secrete glucocorticoids in times of stress in adult depressed individuals with experience of childhood trauma, but the underlying mechanisms are poorly understood (32). A recent study found that the severity of childhood trauma experience contributes to a lack of response to antidepressant treatment and suggested glucocorticoid resistance as a target for the development of personalized treatment for a subgroup of depressed patients with a history of childhood trauma (33). Although our study did not investigate the types of trauma associated with SB among depressed patients, since negative experiences during childhood have everlasting influences (34), we highlight childhood trauma as an emergency.

Some studies have begun to investigate the effects of psychological and social factors on the pathogenic mechanism of SB (35, 36). More than 1/2 of people with suicidal attempts are impulsive, with many suicidal acts conducted with impulsivity (37). Our study also suggests that more attention should be paid to depressed patients with impulsive traits. This group of population is often accompanied by severe executive dysfunction, such as impaired self-inhibition. Importantly, the relationship between executive impairments and suicide risk has been well-established in previous literature (38), suggesting that we should pay attention to the importance of neuropsychological assessment, such as in terms of executive function or impulsiveness. The analysis of functional connectivity in the resting state (RSFC) has been recognized as a powerful well-established technique for unbiased analysis that reveals correlates of activity in discrete brain regions during rest (39) and could provide important insights into intrinsic FC in depressed cases with SB. Cao et al. (40) found that left prefrontal-parietal connectivity was associated with suicidal ideation and levels of impulsivity, but RSFC of the left prefrontal cortex with the right anterior cingulate cortex was correlated exclusively with impulsivity levels and not suicidal ideation in young depressed patients with suicide attempts. Additionally, they argued that the neural circuits underlying suicide attempts might differ from those that underlie suicidal ideation (40). In particular, impulsivity is often confused with agitation in clinical practice (41), and our study did not investigate the effect of agitation on the regression model. Thus, attention should be paid to the assessment of agitation symptoms in future prospective studies.

The severity of depression has been identified as one of the strongest predictors of future suicide attempts (42, 43). In terms of presentation, the severity of depression is often strongly associated with SB in depressed patients, along with other risk factors. For example, Andrewes et al. (44) found that only impulsivity and severity of depression were uniquely predictive of the frequency of suicide attempts. A study conducted in Korea found that 94% of people with suicidal ideation had sleep problems (45). Many studies have found that sleep problems are an independent factor for suicidal ideation, especially short-term sleep (46, 47). However, many studies have found that the relationship between sleep problems and suicidal ideation is mediated by depressive symptoms (45, 48). Hopelessness, worthlessness, helplessness, guilt, and crying are common manifestations of depressive symptoms.

During the last decades, brain imaging techniques provided new approaches to detect structural and functional brain changes *in vivo*, which may help to understand the relationship between depressive symptoms and SB. A systematic retrospective study found that reductions in the volume of basal ganglia and the hippocampus appeared to be more specific for pediatric unipolar disorder, whereas reduced corpus callosum volume and increased rates of deep white matter hyperintensities were more specific for pediatric bipolar disorder (49). Furthermore, the presence of subcortical alterations within the basal ganglia during childhood could subsequently extend to prefrontal cortical regions that continue to develop into adulthood. The prefrontal cortex has been shown to be strongly associated with executive control as impulsivity (50). In addition, a decrease in axonal plasticity in the hippocampus was also found in brain samples of suicide completers (51). As a retrospective case series study, we did not classify depressive symptoms in a multidimensional way. The very important point is that clinicians should pay more attention to these negative emotions, identify high-risk patients early, and then give them timely psychological intervention.

Studies have revealed that susceptibility to suicide among MDD patients is due to the interaction of multiple environmental and biological factors. Suicide is highly familial, and the results of adoption, twin, and family studies suggest that the aggregation of suicide attempts within families is partly due to genetic factors, with heritability estimates of ~40–55% (52). For example, an earlier study concluded that STin2 gene variants and a family history of suicide were significant predictors of suicide completion in depressed cases (53). Additionally, Wang et al. (54) found that a family history of suicide is the most reliable and robust risk factor for suicidal ideation. Neurocognitive deficits have been proposed as an intermediate phenotype between genes and behavioral outcomes for suicide-related behaviors (55). Furthermore, recently a study found that community youth with a family history of suicide performed worse on executive functioning scores, attention tests, and language reasoning (56), suggesting that enhanced neuropsychological testing could contribute to better screening of patients at risk of suicide.

This study may shed some light on clinical practice. Depression patients with the characteristics of the female, with a history of major mental trauma, a family history of suicide, and an impulsive personality, should receive special attention. Currently, clinicians often wrestle with how best to integrate recent advances in suicide prevention into practice in an efficient and effective manner. Suicide risk assessment screening tools are almost uniformly recommended to screen for depressed individuals with suicidal ideation in many countries; however, clinical practice has realistic time constraints, which often makes the predictive power of risk screening tools very limited. In addition to focusing on the risk factors for SB, which could be presented on the Suicide Risk Assessment Scale, Rudd et al. (57) believe that there are four other identifiable aspects that can be addressed in the interview, including the temporal dynamics and natural variability of suicidal ideation and motivation to die, the importance of assessing constructs other than suicidal ideation that are convincingly related to enduring risk or chronic vulnerability to suicide (58–60), the importance of understanding and assessing the potential for poor individual adherence and cooperation with clinical care, and the elegant utility of the expressed wish of patients to live and wish to die, coupled with reasons for living and reasons for dying (57). Due to cultural differences, the form of the interview may vary between physicians in different countries or regions, but the above recommendations help clinicians screen for depressed patients with suicidal ideation in a simple and straightforward manner.

We acknowledge several limitations to this study. First, compared with prospective cohort studies, such electronic medical records-based case series study is associated with certain inevitable drawbacks, including no details on cognitive impairment and education level, and it is suggested that it plays a key role in mediating SB with depression (61, 62). Second, the sample for this study was drawn from a specialist mental health care provider, so it is likely that depressive syndromes are more severe than those determined in the community samples screened. Third, depression diagnoses were based on ICD-10 codes, and there is no information on the procedure by which diagnoses were made. Fourth, the symptoms identified as covariates were derived from those recorded in the text rather than those that could be determined through a screening instrument. Fifth, due to these heterogeneities in sociocultural contexts and sample sources, this study possibly had a certain restricted application in the corresponding fields. Sixth, currently, factors screened by binary logistic regression analysis are independent of each other, the interdependence between them was not examined. Seventh, as a preliminary clinical retrospective study, we did not subdivide SB into suicidal ideation and suicide attempts; this is also the point that we will focus on in the next step.

## Conclusion

Female gender, the history of major mental trauma, impulsivity, the severity of depression, and family history of suicide were independently associated with the appearance of SB among MDD patients in Beijing, China. Inevitably, these findings should be viewed with particular caution due to the inherent drawbacks of a retrospective nature. More prospective longitudinal research should be conducted to examine those dynamic alterations in the corresponding confounders.

## Data availability statement

The raw data supporting the conclusions of this article will be made available by the authors, without undue reservation.

## Ethics statement

The studies involving human participants were reviewed and approved by Beijing Anding Hospital Affiliated to Capital Medical University. Written informed consent for participation was not required for this study in accordance with the national legislation and the institutional requirements.

## Author contributions

CL, WP, and DZ conceived and designed the research protocol. CL completed the data analyses. FM, TT, and LL assisted with data collection and collation. XL revised the manuscript. All authors contributed to the article and approved the submitted version.

## Funding

The present work was supported by the Beijing Municipal Administration of Hospitals Clinical Medicine Development of special funding (ZYLX201815).

## Conflict of interest

The authors declare that the research was conducted in the absence of any commercial or financial relationships that could be construed as a potential conflict of interest.

## Publisher's note

All claims expressed in this article are solely those of the authors and do not necessarily represent those of their affiliated organizations, or those of the publisher, the editors and the reviewers. Any product that may be evaluated in this article, or claim that may be made by its manufacturer, is not guaranteed or endorsed by the publisher.
